# Predictors of a successful vaginal delivery in women with type 1 diabetes: a retrospective analysis of 20 years

**DOI:** 10.1007/s00404-021-06255-9

**Published:** 2021-09-24

**Authors:** Friederike Weschenfelder, Eva Herrmann, Thomas Lehmann, Ekkehard Schleußner, Christof Kloos, Wilgard Battfeld, Tanja Groten

**Affiliations:** 1 Department of Obstetrics, University Hospital Jena, Am Klinikum 1, 07747 Jena, Germany; 2 University Hospital Jena, Institute of Medical Statistics and Computer Science, Friedrich Schiller University, Jena, Germany; 3 Department Internal Medicine III; FB Endocrinology and Metabolic Diseases, University Hospital Jena, Jena, Germany; 4 Medical Care Centre Kempten-Allgäu, Kempten, Germany

**Keywords:** Cesarean section, Diabetes mellitus, type 1, Gestational weight gain, Delivery, Antepartum counseling, Risk factors

## Abstract

**Purpose:**

To evaluate the independent factors associated with the success of a trial of vaginal birth (TVB) in women with type 1 diabetes. Despite all therapeutic efforts and technological innovations, rates of caesarean sections (CS) in pregnant women with type 1 diabetes remain unchanged above 60%. Our aim was to point out influencing factors to improve the quality of antepartum counseling.

**Methods:**

We performed a retrospective cohort study of 195 pregnancies with type 1 diabetes treated between 2000 and 2019. After exclusions, 118 women with near-term singleton pregnancies intended vaginal birth (TVB). Group differences between CS and successful vaginal delivery were analyzed. Multivariate logistic regression was performed by including clinical and metabolic variables to determine the independent effects on a successful vaginal delivery. Subgroup analysis for nulliparous women.

**Results:**

Of 118 women with TVB, 67 (56.8%) were delivered vaginally. History of previous vaginal delivery (OR 10.29; CI 2.39; 44.30), HbA1c changes during pregnancy (per % increase; OR 0.59; CI 0.36; 0.96) and gestational weight gain (per kg; OR 0.87; CI 0.80; 0.96) were independent predictors for a successful vaginal delivery. In nulliparous women, the duration of diabetes was independently and negatively associated with vaginal delivery.

**Conclusion:**

Provided data can help to improve antepartum counseling in type 1 diabetic patients. It seems that women with type 1 diabetes should avoid postponing pregnancy and childbirth.

**Supplementary Information:**

The online version contains supplementary material available at 10.1007/s00404-021-06255-9.

## Introduction

Decades ago, in women with type 1 diabetes pregnancies were rare and highly jeopardized by intrauterine death, preterm delivery, hypertensive disorders or fetal macrosomia. Nowadays, due to new treatment options and substantially improved diabetic control there is a continuous increase of women with type 1 diabetes getting pregnant, reaching term and delivering healthy newborns. Although complications decreased, C-Sections (CS) rates remain high, exceeding 60% [1–5]. As a vaginal delivery is the most desired birth experience in the vast majority of women, this obvious disproportion compared to healthy non-diabetic women is extremely relevant. Women with type 1 diabetes frequently suffer from preexisting diabetes-related complications such as nephropathy and severe hypertension, which consequently obliging elective CS [1, 6]. Thus, higher rates of CS can be explained by higher rates of elective CS. Nevertheless, in those undergoing the trial of vaginal birth (TVB) emergency CS rates are still inexplicably high.

In previous studies nulliparity, hypertensive disorders and previous CS were shown to be significant independent predictors for emergency CS in women with preexisting diabetes [7, 8]. Fischer et al. could not confirm an association with maternal HbA1c-level nor the estimated fetal weight in late pregnancy with the need of an emergency CS [7] while Miailhe et al. showed such association with maternal HbA1c exceeding 6.4% [9]. In the general obstetric population, CS rates are mainly influenced by maternal age, prepregnancy BMI and gestational weight gain (GWG) above recommendations of the institute of medicine (IOM) as well as a history of previous CS [10–15].

Worldwide guidelines for women with pregestational diabetes recommend delivery at 38 to 40 weeks of gestation to prevent the risk of macrosomia and still birth. This management results in a high number of women who undergo induction of labor (IOL) [1, 4]. IOL is controversially discussed as a potential risk factor for emergency CSs [16, 17].

Available cohort studies investigating obstetric outcome in women with preexisting diabetes did so far not specifically focus on the cohort aspiring vaginal birth. In these studies, no differentiation between elective and emergency CS was made. In our study, we specifically focused on predictors of successful vaginal delivery in women with TVB. Our aim was to detect factors associated with the success of TVB to improve the quality of antenatal counseling of women with type 1 diabetes.

## Methods

### Study population

**Fig. 1:**
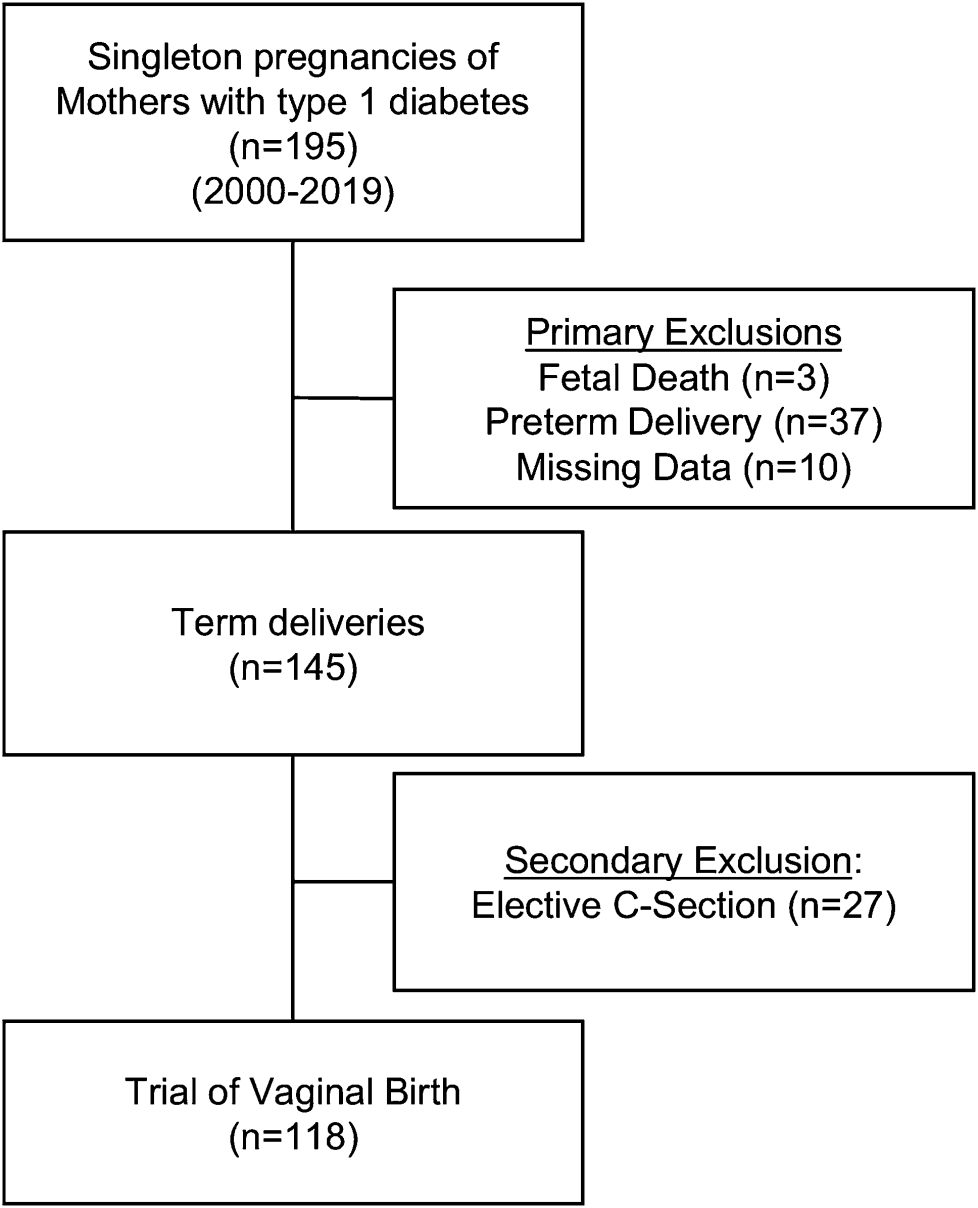
Cohort composition—The final cohort consists out of 118 women with type 1 diabetes and trial of vaginal birth (TVB). 195 pregnancies with type 1 diabetes were treated in our outpatient clinic between 2000 and 2019. All cases of stillbirths (*n* = 3), preterm deliveries (< 37 weeks of gestation, *n* = 37) and cases with incomplete data (*n* = 10) and elective caesarean sections (CS; *n* = 27) were excluded

The study cohort consists of 118 women with type 1 diabetes treated at our outpatient department for diabetes and pregnancy. We included singleton type 1 diabetes pregnancies, with term deliveries (≥ 37 weeks of gestation) and TVB consulted from 1st January 2000 until 31st December of 2019 as shown in Fig. 1. Diabetes care was applied according to German guidelines [18] and provided by our hospital-based outpatient department. Ethical approval was given by the local Ethical Committee of the Friedrich-Schiller-University, Jena, Germany (2021–2159, date of re-approval 19.03.2021).

### Study data collection

Basic characteristics and patient’s history were retrieved from patient records. Duration of diabetes was defined as the time range between the first diagnosis of type 1 diabetes and first patient contact during pregnancy in our outpatient clinic. Insulin treatment methods during pregnancy were stratified in multiple daily injections (MDI) or continuous subcutaneous insulin injections (CSII, pump therapy) regardless of the method used before the pregnancy.

The prepregnancy BMI was calculated from maternal height and the documented prepregnancy weight and further categorized according to the definitions of the world health organization (WHO) [19]. GWG was defined as the difference of the prepregnancy weight and the last documented weight during pregnancy and categorized according to IOM criteria in “recommended GWG” vs. “excessive GWG” depending on prepregnancy BMI classes. “Recommended GWG” included all cases that showed a GWG below or within the range of recommended weight gain. “Excessive GWG” was used in case IOM recommendations had been exceeded [15]. ‘Early pregnancy HbA1c level’ was defined as first documented Hb1c level in the records and ‘HbA1c level at delivery’ was defined as the last documented HbA1c level determined during pregnancy. Measurements up to a maximum of four weeks before delivery were included. HbA1c was measured according to IFCC or NGSP/DCCT standard. HbA1c changes were calculated using the difference between ‘early pregnancy HbA1c levels’ and ‘HbA1c level at delivery’ (in %). Birth history data included number of pregnancies, number of deliveries and mode of delivery, distinguishing between the history of CS and any type of vaginal delivery. Perinatal outcome data retrieved from the standardized nationwide used perinatal documentation systems of our University hospital and patient’s maternity records included: IOL, mode of delivery, gestational age at delivery, fetal birth weight, 5-min Apgar score, postnatal neonatal intensive care unit (NICU) admission and neonatal hypoglycemia and hyperbilirubinemia. Fetal birth weight was categorized using Voigt’s percentiles for the body measurement of newborns and defined large for gestational age (LGA) above 90th percentile and small for gestational age newborns (SGA) below 10th percentile adjusted for gestational age and fetal sex [20].

### Patient monitoring

Most women with type 1 diabetes are seen in the first trimester of their pregnancy at our obstetric outpatient clinic. Routine maternity and diabetes care continued to be provided by the women’s respective diabetes and obstetric specialists. At the first visit, status of diabetic control is evaluated, specific details of a pregnancy with type 1 diabetes are discussed, relating information is provided and further appointments depending on the severity and needs of each individual case are planned. In cases of uncomplicated pregnancies, women are scheduled for at least two more appointments around 28–30 weeks of gestation and around 34–35 weeks of gestation for their birth planning consultation. During that consultation, based on obstetric history, estimated fetal weight, medical history and glycemic control, time point, and mode of delivery are selected by informed consent. The aim here is to empower women with type 1 diabetes to deliver vaginally, whenever possible and the women want it. If women do not go into labor spontaneously, induction of labor is scheduled at the estimated date of delivery according to international guidelines or earlier in case of upcoming complications. Induction of labor was usually performed using double-balloon catheter for cervical ripening followed by oral or vaginal misoprostol if suitable.

### Statistical analysis

Statistical analysis was performed with SPSS 24.0. No prior sample size estimation was performed. The Chi^2^ test or Fisher exact test was used to compare categorical data. Most of the continuous data were not normally distributed; therefore, our data are presented using median and interquartile range. Nonparametric tests were used to compare continuous data between the two subgroups: vaginal delivery and CS. Benjamini–Hochberg correction was used for controlling the familywise error rate due to multiple testing [21]. Adjusted odds ratios (ORs) for estimating the association between vaginal delivery and duration of diabetes, maternal age, history delivery, prepregnancy BMI, GWG, CSII, HbA1c changes, fetal birth weight and gestational age at delivery were determined using logistic regression. Variables included in the model were chosen a priori. Generalized estimating equations were used to prove that there is no effect due to repeated observations because of multiple deliveries of one individual in the entire cohort. ORs with 95% confidence interval (CI) are presented. A *p* value < 0.05 was considered to indicate statistical significance (2-tailed).

## Results

Table 1 shows maternal characteristics, pregnancy—and neonatal outcome. Of 118 women with type 1 diabetes and TVB 67 (56.8%) women delivered vaginally und 51 (43.2%) had to undergo emergency CS. Causes for emergency CS were fetal distress (54.7%, *n* = 28), failure to progress and cephalopelvic disproportion (31.4%, *n* = 16); maternal (3.9%; *n* = 2), fetal malpresentation (2%, *n* = 1) and four cases with other indications (7.8%). Reasons for primary CS were preexisting diabetic complications, fetal macrosomia (estimated fetal weight > 4500 g), previous CS and breech positions (Data not shown).

**Table 1:** Main characteristics of women with type 1 diabetes and intended vaginal delivery in term singleton pregnancies (TVB; *n* = 118) and the subgroup comparisons for vaginal delivery (VD; *n* = 67) and C-Section (CS; *n* = 51)

Variable	Entire cohort (*n* = 118)	VD (*n* = 67)	CS (*n* = 51)	*p*
Maternal baseline characteristics				
Maternal age (years)	29 (26;33)	28 (26;32)	30 (27;33)	0.197
Duration of diabetes (years)	13 (7;20)	11 (5;16)	16 (9;22)	0.008^†^
Number of pregnancies	1 (1;2)	2 (1;2)	1 (1;2)	0.071
Nulliparous	66.1%	52.2%	78.4%	0.004*
History of CS	11.9%	9%	15.7%	0.001*
Prepregnancy weight (kg)	67 (59;74)	69 (60;76)	66 (58;70)	0.329
Prepregnancy BMI (kg/m^2^)	24.3 (21.2;26.5)	24.3 (21.3;26.5)	24.2 (21.2;26.8)	0.881
Obesity (BMI ≥ 30 kg/m^2^)	7.6%	4.5%	11.8%	0.383
CSII	67.8%	59.7%	78.4%	0.046^†^
Preexisting diabetic complications (*n* = 100)	14%	11.5%	17.9%	0.389
Pregnancy outcome				
GWG (kg)	16 (11.7;19)	14 (10.5;17.2)	17.3 (14.5;22)	0.002*
Excessive GWG (%)	59.3%	50.7%	70.6%	0.038
Early pregnancy HbA1c (in mmol/mol)	45 (39;53)	48 (39;55)	43 (37;51)	0.134
Early pregnancy HbA1c (in %)	6.3 (5.7;7.0)	6.5 (5.7;7.2)	6.1 (5.5;6.8)	
Hb1c level at delivery (in mmol/mol	39 (34;44)	40 (34;45)	38 (36;43)	0.773
Hb1at delivery (in %)	5.7 (5.3;6.2)	5.8 (5.3;6.3)	5.6 (5.4;6.1)	
HbA1c changes (in mmol/mol)	− 6 (− 13;1)	− 7(− 14; 0)	− 3 (− 12; 2)	0.303
HbA1c changes (in %)	− 0.5 (− 1.2;0.1)	− 0.6 (− 1.3–0)	− 0.3 (− 1.1;0.2)	
Max. insulin dose/kg/day	0.84 (0.62;1.0)	0.79 (0.65;1.0)	0.88 (0.61;1.1)	0.378
Pre-eclampsia/PIH/HELLP	9.4%	9.1%	9.4%	1.0
IOL	70.4%	63%	79.5%	0.81
GA at delivery (weeks)	38 (38;39)	38 (37;39)	38 (38;39)	0.617
CS	43.2%	;	100%	
Shoulder dystocia (*n* = 80)	2.5%	5.3%	;	
Neonatal outcome				
Male/female newborn	45.8%/54.2%	41.8%/58.2%	51%/49%	0.355
Birth weight	3650 (3339;3960)	3650 (3350;4000)	3580 (3290; 3820)	0.203
LGA	23.7%	28.4%	17.6%	0.197
SGA	3.4%	3%	3.9%	1.00
Voigt’s percentile	78 (47;90)	79 (48;93)	75 (45;86)	0.178
Ponderal index percentile	72 (40;95)	76 (40;96)	71 (38;95)	0.845
5 min APGAR	9 (8;10)	9 (8;10)	9 (8;10)	0.884
pH	7.22 (7.16;7.26)	7.21 (7.14;7.25)	7.24 (7.17;7.27)	0.67
NICU > 2 days (*n* = 99)	42.4%	43.1%	41.7%	1.00
Hyperbilirubinemia (*n* = 92)	31.5%	37.5%	22%	0.168
Hypoglycemia (*n* = 105)	38.1%	32.8%	44.7%	0.231

Data are percent or median and interquartile range (IQR) unless otherwise specified

*BMI* body mass index, *CSII* continuous subcutaneous insulin infusion, *CS* cesarean section, *GA* gestational age, *GWG* gestational weight gain, *IOL* induction of labor, *LGA* large for gestational age, *;* odds ratio, *NICU* neonatal intensive care unit, *PIH* pregnancy-induced hypertension, *SGA* small for gestational age, *VD* vaginal delivery

*Remaining significant differences (*p* < 0.05) after using Benjamini–Hochberg correction: Nulliparous (*p* = 0.04), History of CS (*p* = 0.03) and GWG (*p* = 0.03)

^†^Not significant (*p* > 0.05) after using Benjamini–Hochberg correction for multiple testing

The groups did not differ concerning maternal age, number of preceding pregnancies, preexisting diabetic complications (e.g. retinopathy, nephropathy) prepregnancy weight, prepregnancy BMI and obesity rates. Women delivered by emergency CS were more likely to be nulliparous 78.4% vs. 52.2% (*p* = 0.04) and had significantly higher rates of previous CS 15.7% vs. 9% (*p* = 0.03). Duration of diabetes (16 years vs. 11 years) and the number of patients on CSII (78.4% vs. 59.7%) showed a strong trend towards the failure of TVB but failed to reach statistical significance after using correction for multiple testing (*p* = 0.06).

Women succeeding in vaginal delivery had a significantly lower GWG with 14 kg (IQR 10.5; 17.2) compared to women with CS 17.3 kg (IQR 14.5; 22) (*p* = 0.03). Accordingly, more women with emergency CS showed excessive GWG according to IOM recommendations: 70.6% vs. 50.7% (but, *n*.s.). Both groups showed no differences in perinatal outcomes and HbA1c levels.

Concerning the neonatal outcome, we could not find differences in birth weight, ponderal index, LGA and SGA status, 5 min APGAR; umbilical cord pH, fetal sex, hyperbilirubinemia, NICU admission (> 2 days) or, hypoglycemia.

Multivariate analysis revealed history of vaginal delivery (OR 10.3; CI 2.39; 44.30), GWG (per kg; OR 0.87; CI 0.80; 0.96) and HbA1c increase (per %, OR 0.59; CI 0.36; 0.96) to be significantly and independently associated with a successful vaginal delivery. (Table 2) In the subgroup analysis of nulliparous women (*n* = 75), duration of type 1 diabetes (per year, OR 0.91; CI 0.84; 0.99) and GWG (per kg, OR 0.90; CI 0.81; 0.99) were negatively and independently associated with a vaginal delivery.

**Table 2:** Factors associated with successful vaginal delivery in women with type 1 (*n* = 118) and subgroup of nulliparous women (*n* = 75)

Groups	Variables	OR	CI	*p*
Entire Cohort (*n* = 118)	Duration of diabetes (years)	0.95	[0.88; 1.03]	0.382
	Maternal age at delivery (years)	0.92	[0.84; 1.01]	0.085
	History of Vaginal Delivery*	10.29*	[2.39; 44.30]	0.002
	History of CS	1.09	[0.28; 4.24]	0.897
	Prepregnancy BMI (kg/m^2^)	0.90	[0.79; 1.02]	0.104
	GWG (kg)*	0.87*	[0.80; 0.96]	0.003
	CSII	0.64	[0.21; 1.96]	0.434
	HbA1c change^†^ (%)*	0.59*	[0.36; 0.96]	0.035
	Fetal birthweight (g)	1.00	[1.00; 1.00]	0.055
	GA at delivery (weeks)	0.87	[0.54; 1.40]	0.569
Nulliparous (*n* = 75)	Duration of diabetes (years)*	0.91*	[0.84; 0.99]	0.032
	Maternal age at delivery (years)	0.88	[0.78; 1.00]	0.050
	Prepregnancy BMI (kg/m^2^)	0.95	[0.81; 1.12]	0.552
	GWG (kg)*	0.90*	[0.81; 0.99]	0.046
	CSII	0.79	[0.25; 2.49]	0.685
	HbA1c change^†^ (%)	0.76	[0.39; 1.47]	0.412
	Fetal birthweight (g)	1.00	[1.00; 1.00]	0.952
	GA at delivery (weeks)	0.86	[0.47; 1.56]	0.622

*BMI* body mass index, *CI* confidence interval, *CSII* continuous subcutaneous insulin infusion, *GA* gestational age, *GWG* gestational weight gain, *OR* odds ratio

^*^Significant independent variables (*p* < 0.05)

^†^HbA1c change between baseline and delivery in %

## Discussion

### Main findings

Aim of this study was to find parameters affecting the success of vaginal delivery in women with type 1 diabetes to provide profound knowledge or antepartum counseling. History of previous vaginal delivery, glycemic control and GWG revealed to independently impact vaginal delivery. The latter two are especially important, as they can be influenced by pregnancy management and the patient herself. A precondition to improve self-management is to provide essential information by the care provider. Additionally, subgroup analysis of nulliparous women showed that the duration of diabetes independently affected the chance of vaginal delivery, a fact that needs to be mentioned to young diabetic women.

### Strengths and limitations

Main limitations of this study are the retrospective, unicentric design and the general reliability of electronic records. An obvious strength is the quality and quantity of detailed data of a relatively large cohort of pregnant women with type 1 diabetes. Furthermore, the rather high percentage of women undergoing TVB in our cohort is considerably notable. The equally distributed baseline characteristics between the two groups (vaginal delivery vs. CS) constitutes an additional strength of our analysis. The number of repeated deliveries in one individual may give a general limitation in cohorts reporting a study period of 20 years. We minimized the effect by using Generalized Estimated Equations models for the statistics. The length of the study period may also have had an impact on the outcomes observed, since therapy regimes improve continuously over time. The effect of IOL on the mode of delivery cannot be judged in this study, since we were not able to include information on IOL in the regression model due to statistical limitations.

### Interpretations

In our cohort of 118 women with type 1 diabetes and TVB, 56.8% gave birth vaginally. From the initial 145 term deliveries, 27 women had an elective CS (18.6%). The overall CS rate including elective and emergency CS in this cohort is 53.8% which is nearly twice as high as the overall CS rate in Germany with 32% [22]. Although not satisfying, this CS rate is lower than reported by others in diabetic pregnancies. The lowest so far documented rates are 60% in a corresponding cohort of women with type 1 diabetes treated at another German University Hospital and 60.55% reported by Metcalfe and coauthors in a large Canadian cohort [2, 23].

In nulliparous women, diabetes duration remained an independent negative predictor for vaginal delivery, revealing a reduction in success to deliver vaginally of 8.7% for each extra year of diabetes duration in this subgroup (see Supplemental Table S1). Interestingly, this was not associated with a difference in preexisting diabetic complications. We could not confirm this effect of diabetes duration in the entire cohort. Neither did Lepercq et al. in their study on the determinants of a combined good perinatal outcome (including vaginal delivery) in type 1 pregnancies [24]. Both groups were comparable concerning maternal age and prepregnancy BMI.

To our surprise, we observed a higher rate of CSII in the group resulting in CS (78.4%). Possibly, the association of CSII with an increased risk for CS might be explained by the accompanied increase in GWG [17.3 kg (14.5–22)] in this group. Consistently, Hauffe et al. also showed that women with CSII gained more weight during pregnancy compared to women on MDI treatment. The authors discussed higher compensatory carbohydrate intake due to more frequent hypoglycemia as well as more intake due to dietary flexibility [2] as potential reasons for this observation. Conclusively, previous studies have shown that higher GWG is associated with an increase in CS rates. Exceeding IOM recommendations were significantly associated with a 1.3–1.4-fold higher risk to undergo emergency CS as shown by Nilses et al. and Goldstein et al. [10, 25]. In our cohort, 70.6% women with CS gained weight above the recommendations compared to 50.7% in the group with vaginal delivery. Consequently, GWG remained an independent influence factor of a successful vaginal delivery in the multivariate analysis. Therefore, women with type 1 diabetes need counseling to monitor their GWG from the beginning of their pregnancy. Especially women with CSII need to be aware of their specific risk of more GWG. Hauffe et al. also found a higher rate of LGA infants in the CSII group possibly mediated by excessive GWG [2].

In our cohort, LGA rates were 23.7% and thus twice as high as in the general population with rates about 10%. However, the rate of 23.7% LGA born was remarkably lower in our cohort compared to other studies reporting LGA rates exceeding 30 or even 40% in women with type 1 diabetes [2, 26].

Metcalfe et al. showed that 40.56% of the neonates of diabetic mothers had a prolonged admission to NICU [23]. These results correspond with our findings of 42.4% of the entire cohort who stayed at NICU for more than two days. However, we did not find a significant difference between vaginal und CS deliveries. The comparable high number of NICU admissions in our cohort is partially explained by internal clinical standards recommending NICU admission for all newborns of women with pregestational diabetes regardless their clinical status until 2014.

Elevated BMI is a well-known risk factor for adverse perinatal outcome including emergency CS in all women. Previous studies also proved this effect in women with type 1 diabetes [27]. In our cohort, only 7.6% women were obese: 4.5% in the vaginal delivery group and 11.8% in the CS group. The median prepregnancy BMI was ~ 24 kg/m^2^ in the cohort as well as in all groups, representing a rather lean cohort. We think that the narrow range of BMI values could be a reason for the missing impact on perinatal outcome in our study cohort.

Not surprising was the fact that multivariate regression revealed that history of vaginal delivery is an independent positive predictor of successful vaginal delivery (OR 10.29; CI 2.39–44.30). Remarkably, the history of CS did not independently affect vaginal delivery.

It is well known that diabetic control influences perinatal outcomes. In the study of Lepercq et al. they showed a continuous relationship between HbA1c at delivery and a good perinatal outcome defined as uncomplicated term delivery without macrosomia and lack of neonatal morbidity [24]. Nevertheless, they did not prove an independent association of HbA1c level with CS [27]. We included HbA1c change during pregnancy, rather than pure HbA1c value at the time of birth, in our multivariate analysis and could demonstrate that an increase of HbA1c levels by each 1% lowered the chance of a vaginal delivery to 58.9% compared to their overall chances. Resulting in halving the probability of a vaginal delivery with each additional increase in percentage of HbA1c for each women. Successful managing of glucose control during pregnancy therefore will also improve outcome regarding the mode of delivery.

## Conclusion

In this descriptive study, factors that predict the success of vaginal delivery were history of vaginal delivery and the modifiable factors diabetic control and GWG. For nulliparous women, the duration of diabetes seems to be of special interest—postponing pregnancy and childbearing should therefore be avoided. Our study provides data, which can improve the quality of counseling pregnant women with type 1 diabetes regarding what to expect pursuing vaginal delivery.

## Supplementary Information

Below is the link to the electronic supplementary material.

Supplementary file1 (DOCX 19 KB) Supplemental Table S1 Main characteristics of nulliparous women with type 1 diabetes an intended vaginal delivery in term singleton pregnancies (TVB; n=75) and the subgroup comparisons for vaginal delivery (VD; n=35) and caesarean section (CS; n=40)

## Data Availability

The datasets used and/or analyzed during the current study are available from the corresponding author on reasonable request.
