# Supplementary material for: Predictors of a successful vaginal delivery in women with type 1 diabetes: a retrospective analysis of 20 years
Source: Arch Gynecol Obstet. 2021 Sep 24;305(6):1445–52. doi: 10.1007/s00404-021-06255-9 (PMC9166876; doi:10.1007/s00404-021-06255-9)
Supplement: Supplementary file 1 — Supplementary file1 (DOCX 19 KB) Supplemental Table S1 Main characteristics of nulliparous women with type 1 diabetes an intended vaginal delivery in term singleton pregnancies (TVB; n=75) and the subgroup comparisons for vaginal delivery (VD; n=35) and caesarean section (CS; n=40) [file 404_2021_6255_MOESM1_ESM.docx]

Supplemental Table S1 - Main characteristics of nulliparous women with type 1 diabetes an intended vaginal delivery in term singleton pregnancies (TVB; n=75) and the subgroup comparisons for vaginal delivery (VD; n=35) and C-Section (CS; n=40)

| Variable | Nullipara  (n=75) | VD  (n=35) | CS  (n=40) |  |
| --- | --- | --- | --- | --- |
| Maternal Baseline Characteristics | |  |  | p |
| Maternal age (years) | 28 (25-32) | 27 (23-29) | 30 (26-33) | .01† |
| Duration of diabetes (years) | 12 (7;19) | 10 (4-15) | 16 (9-22) | .001* |
| Prepregnancy weight (kg) | 66 (58-72) | 70 (59-74) | 64.9 (58-70) | .617 |
| Prepregnancy BMI (kg/m²) | 23.8 (20.7-26.1) | 24.2 (20.6-26.1) | 23.8 (20.8-26.6) | .865 |
| Obesity (BMI > =30 kg/m^2^) | 5.3% | 0% | 10% | .232 |
| CSII | 66.7% | 57.1% | 75% | .141 |
| Preexisting diabetic complications | 14.5% | 12.1% | 17.2% | .722 |
| Pregnancy Outcome | |  |  |  |
| GWG (kg) | 16.4 (11.9-19) | 15.7 (10.5-17.7) | 17.3 (14.6-20.6) | .025† |
| Excessive GWG (%) | 59.3% | 50.7% | 70.6% | .038† |
| Early pregnancy HbA1c level (in %) | 6.1 (5.5-6.9) | 6.1 (5.5-7.0) | 6.1 (5.4-6.8) | .592 |
| (in mmol/mol) | 43 (37-52) | 43 (37-53) | 43 (36-51) |  |
| Hb1c level at delivery (in %) | 5.6 (5.4-6.0) | 5.7 (5.4-6) | 5.6 (5.4-6) | .774 |
| (in mmol/mol) | 38 (36-42) | 39 (36-42) | 38 (36-42) |  |
| HbA1c changes (in %) | -.5 (-1.1-.2) | -.5 (-1.1-.1) | -.2 (-1.2-.2) | .573 |
| (in mmol/mol) | -5.5 (-12.1-2.2) | -5.5 (-12.1-1.1) | -2.2 (13.2 -2.2) |  |
| Max. insulin dose /kg/day | .86-(.63-1.11) | .81 (.65-1.15) | .90 (.62-1.10) | .803 |
| Pre-eclampsia/ PIH/ HELLP | 10.8% | 11.8% | 10% | 1.0 |
| IOL | 73.8 | 65.4% | 80% | .246 |
| Gestational age at delivery | 38 (38-39) | 38 (37-39) | 38 (38-39) | .663 |
| Shoulder dystocia | - | - | - |  |
| Neonatal Outcome | |  |  |  |
| Male/Female Newborn | 45.3%/54.7% | 40%/60% | 50%/50% | .487 |
| Birth weight | 3560 (3300-3820) | 3530 (3250-3715) | 3630 (3312-3891) | .323 |
| LGA | 17.3% | 14.3% | 30% | .557 |
| SGA | 4% | 2.9% | 5% | 1.0 |
| Voigt’s Percentile | 72 (42-84) | 66 (41-82) | 75.5 (44.3-86) | .407 |
| Ponderal Index Percentile | 64 (35-94) | 61 (31-94) | 67.5 (39.3-95) | .497 |
| 5min APGAR | 9 (8-10) | 9 (8-10) | 9 (8-10) | .421 |
| pH | 7.21 (7.15-7.26) | 7.18 (7.12-7.22) | 7.24 (7.17-7.27) | .017† |
| NICU > 2 days | 44.1% | 45.5% | 43.2% | 1.0 |
| Hyperbilirubinemia | 26.8% | 32.1% | 21.4% | .547 |
| Hypoglycemia | 41.5% | 37.9% | 44.4% | .623 |

Data are percent or median and interquartile range (IQR) unless otherwise specified.

* only remaining significance (p=.027) after using Benjamini-Hochberg correction for multiple testing

† not significant (p>.05) after using Benjamini-Hochberg correction for multiple testing

BMI-body mass index; CSII – continuous subcutaneous insulin infusion; CS- cesarean section; GA – gestational age; GWG – gestational weight gain; IOL – induction of labor; LGA – large for gestational age,; – odds ratio; NICU – neonatal intensive care unit; PIH – pregnancy induced hypertension, SGA – small for gestational age; VD – vaginal delivery
